# A Modified Operation in Excision of the Os Maxillare Superius

**Published:** 1850-01

**Authors:** W. E. Horner

**Affiliations:** Professor of Anatomy in the University of Pennsylvania, and Senior Surgeon in the St. Joseph’s Hospital


*A Modified Operation in Excision of the Os Maxillare Superius.* By W. E. Horner, M. D., Professor of Anatomy in the University of Pennsylvania, and Senior Surgeon in the St. Joseph's Hospital.

This once formidable operation, has latterly been so often performed successfully, that but little of novelty, or of special interest, is now attached to it. Its feasibility thus established, a mitigated form of its execution may, however, be a sufficient excuse for the present brief allusion to a case of the kind.

The following letter from the patient himself, exhibits the early condition and progress of his affection. He is now aged eighteen.

Dr. Horner: Dear Sir,—In August, 1847, I suffered for four or five days from a severe headache, sickness of stomach, and a general uncomfortable feeling, which was followed by a purulent discharge from a small orifice in the left side of the roof of the mouth. After a few days, two or three pieces of a hard substance sloughed out, leaving an opening the size of a pea-nut. Soon after this, the discharge ceased, and the opening gradually healed up. At this time there was a flat tumour occupying nearly the whole roof of the mouth on that side. This swelling increased slowly, and extended so as to involve the alveolar portion of the maxillary bone, and cause a loosening of the back teeth. About the middle of last May, the middle molar tooth was extracted, without, however, causing any material difference in the size or appearance of the tumour; which, during the few months previous to its removal, increased, I think, somewhat more rapidly than in its earlier stages. From the time of the first appearance of the tumour, to its removal, I am not conscious of having suffered any pain from it.

Yours respectfully, M. W. A.

In consultation with Drs. J. M. Allen, the uncle of the patient, George W. Norris, and Henry H. Smith, a decision was had for the removal of the diseased bone along with the tumour.

During the deliberations on the case, which lasted two or three weeks, the tumour had grown somewhat, but was not painful; its size, however, was inconvenient in eating and talking, and affected the motions of the tongue. There had been no discharge from the nose, which led us to hope that the tumour had not reached the antrum highmorianum.

The operation occurred on the 28th day of October, with the assistance of the surgeons above named, and some young gentlemen, students of medicine. The tumour, at this date, occupied the whole of the left side of the hard palate, and made a volume of about half the size of the largest hen egg. All the teeth of the left upper jaw were loose, the posterior ones rising from their sockets, and the anterior vibrating sensibly. It was thus plain that the gums and alveoli were all affected by the extension of the disease.

I had previously determined to avoid, if possible, the through incision of the cheek as commonly practised, owing to the permanent deformity left by it. The following proceeding was therefore had. The patient, seated in a chair, was placed under the influence of ether, by inhalation. The mouth being then held open, the upper lip and the cheek were raised from the maxilla superior, by an interior incision in a line parallel with the superior margin of the buccinator. This part of the operation was preceded by the extraction of the two left incisor teeth. The corresponding alveoli were then removed. The first step of the latter was to cut in front, with a narrow saw, along the middle palate suture from the mouth into the left nostril, until the palate plate was reached; then, with a pair of strong hawk-bill scissors, such as are used by gardeners for lopping off twigs, an incision was made so as to take out at a clip the whole of the two vacated alveoli, in other words, what is considered by some as the intermaxillary bone of the human subject. A thin, flat knife, well tempered, and with a strong round handle, was then passed from the mouth into the nose, at the posterior part of the middle palate suture, and this suture cleaved in its length from the soft palate to the nick left by the excision of the intermaxillary bone. The narrow saw was again used to cut through the root of the nasal process of the maxillare superius. A pair of strong scissors, curved on the flat, was then taken, to clip through the exterior and the interior side of the maxillare superius, a little below the orbitar plate; the incision being carried back to the pterygoid process of the sphenoid bone. The base of the soft palate was then detached by a short triangular knife, curved on the flat. The greater part of the upper maxilla being thus loosened all around, and insulated, it was brought away with the tumour all in a body, after cutting through a few shreds of attachment to the back of the cheek. The pterygoid process—the os malæ, and the orbitar plate of the maxillare superius, were not disturbed, but left.

The tumour, contrary to our hopes, also occupied the antrum, so as to be as large above as it was in the mouth; it was also attached to the posterior part of the cheek, and to the external pterygoid muscle. A careful removal of it with gouge and scissors, was accomplished wherever any part of it could be detected.

The bleeding was profuse, especially from what was considered to be the posterior palatine artery; it was secured with the aid of Physick's forceps and needle; some few other arteries were also secured by ligature. The parts being well washed, were then carefully packed with successive small pellets of coarse charpie, firmly pressed in, and the base of the packing was supported by a thin board of a semicrescentic shape, somewhat larger than a half of the hard palate. This board was sustained by the teeth of the lower jaw, the latter being fixed by a bandage as in fracture.

The bleeding in this case was so profuse during the operation, that the latter was suspended at intervals, so as to clear the throat from the accumulation of blood, which embarrassed respiration.

The dressing was so effectual, that we had no trouble with the bleeding afterwards. By the 9th day of November, i. e. in twelve days, the patient was in a fine state; the lint and the ligatures had all come away. In a few days afterwards, the patient began to exercise out of doors, and by the last of the month, I had the pleasure of seeing him apparently perfectly well, with not the slightest external indication of the operation, and with a face so straight that his nearest acquaintance would not, of himself, have suspected any thing, and least of all, that almost the whole of the upper maxillary bone had been excised.

The board is liable to displacement; I should, therefore, under similar circumstances again have a spring, or some arrangement to fix it permanently and securely.

The additional time required for this mode of operating, is probably fifteen or twenty minutes; but as a scar in the face is an affair for life to the patient, it should, if possible, be avoided, which, if it cannot be accomplished in all, may certainly be in many cases.

